# How is intergenerational emotional cohesion linked to depression among older internal migrants in China: the mediating roles of loneliness and perceived stress

**DOI:** 10.1186/s40359-024-01579-y

**Published:** 2024-02-23

**Authors:** Ruyue Deng, Shiyuan Yan, Lin Zhang, Yanjie Hou, Hao Wang, Wenjing Zhang, Jun Yao

**Affiliations:** 1 School of Health Policy and Management, Nanjing Medical University, Nanjing, China; 2 School of Nursing, Nanjing Medical University, Nanjing, China; 3 Institute of Healthy Jiangsu Development, Nanjing Medical University, Nanjing, China

**Keywords:** Older internal migrants, Intergenerational relationships, Emotional cohesion, Depression, Loneliness, Perceived stress

## Abstract

**Background:**

Late-life internal migration is frequently associated with a higher risk of depression in older parents. This research delves into the impact of intergenerational emotional cohesion (IEC) on depression in older internal migrants and the underlying mechanisms within the contemporary Chinese context.

**Methods:**

Obtained from a cross-sectional survey in Nanjing, China, the research involved 654 older internal migrants (66.97% female; mean age = 66.05 years; SD = 4.67). Variables were assessed using the Intergenerational Solidarity Inventory, 3-item R-UCLA Loneliness Scale, Perceived Stress Scale, and 9-item Patient Health Questionnaire (PHQ-9). For mediation exploration, a serial mediation model was utilized, and the Bootstrap method was employed to test the signifcance of these mediation effects.

**Results:**

IEC demonstrates a negative correlation with depression. Through IEC, three significant mediation pathways were identified that directly affect depression: (1) loneliness (β=-0.06; Ratio=17.14%), (2) perceived stress (β=-0.09; Ratio=25.71%), and (3) loneliness and perceived stress (β=-0.03; Ratio=8.57%).

**Conclusions:**

IEC can impact the depression of older internal migrants by mitigating negative psychological emotions during the migration process. This finding provides valuable theoretical insights for the prevention of mental health problems among this demographic.

## Background

Propelled by the dual forces of urbanization and the aging demographic in China, internal migration in late life has emerged as a noteworthy phenomenon and tendency [1]. Driven by the motivation of family reunification and the responsibility of caring for grandchildren [2–4], an increasing number of older parents have relocated from their hometowns to urban centres where their adult children live and work [1, 2]. According to the seventh Chinese census, the number of internal older migrants aged 60 or above reached 33.27 million in 2020, marking a 2.14-fold increase from the figures in 2010 [2, 5]. Late-life migration, whether it’s international or internal, and the migration-related processes have consistently been linked to a heightened risk of depression among older people. This pattern is observed in both Western and Eastern samples [6–9]. Existing Chinese literature indicates that older internal migrants face a greater risk of depression compared to their local counterparts [10, 11]. For instance, a study on older internal migrants in China revealed that approximately 34.90% experienced mild or severe depression [12]. Therefore, it is imperative to investigate the factors determining depression in older internal migrants within the Chinese context.

In China, enduring Confucian traditions underscore collectivist familism and filial piety [13]. Consequently, Chinese older parents tend to place a high value on intergenerational relationships and family cohesion [14]. According to the Social Convoy Model, social relations provide support to individuals at different stages of life, enabling them to cope with stress and promoting their physical and mental well-being [15]. Typically, the convoy structure is depicted as concentric circles, symbolizing different levels of closeness and social support [16]. For older parents, close adult children, who often provide the highest levels of support and affection, are positioned in the innermost circle [17]. Researchers have discovered that intergenerational relationships with adult children, particularly in terms of emotional closeness, play a pivotal role in mitigating depressive symptoms among Chinese older parents [14, 18, 19]. However, China’s profound social changes and experiences of internal migration in late life have reshaped family structures, weakened family authority, and intensified intergenerational conflicts [20, 21], posing challenges to the intergenerational relationships of older Chinese internal migrants. Hence, this study sought to explore the impact of intergenerational relationships on depression among older internal migrants and the underlying mechanisms in the contemporary Chinese context.

### Older internal migrants in the Chinese context

Based on the cultural virtues of filial piety and familial care, this group of older migrants manifests distinctive migration patterns, motivations, challenges, and needs [1, 4]. Firstly, unlike older migrants in developed nations and regions who frequently move to more favourable climates or cost-effective areas for an improved retirement lifestyle [22], the migration pattern of Chinese older parents is notably influenced by the migration behaviour of their adult children [2]. Family reunion and participation in grandparenting have become the primary motivations for the migration of older parents [1, 3, 4]. Moreover, the Hukou System (Household Registration System, HRS), a distinct urban-rural segregation in China, presents an adaptation barrier. Under this constraint, hukou, along with the associated social and welfare rights (i.e., social security, community eldercare services, and public health services), does not transfer with older migrants within the country [23–25]. Hence, this distinctive Chinese context makes it increasingly important to comprehend the mechanisms and conditions under which intergenerational relationships impact the depression of older internal migrants.

### Intergenerational emotional cohesion and depression

Intergenerational Emotional Cohesion (IEC) stands as a core dimension of intergenerational relationships, encompassing positive emotional connections between older parents and adult children [26]. In this study, we lean upon the intergenerational solidarity framework to conceptualize IEC [27]. It includes sentiments of affection, emotional closeness, and emotional support, constituting the pivotal dimension of the framework [28]. Confirmed by prior research in China, intergenerational relationships exert a positive influence in mitigating depressive symptoms among older adults [14, 18, 19, 29, 30]. Notably, among all dimensions of intergenerational relationships, IEC plays the most significant role [14, 19, 29]. Similar results have been identified in studies involving older internal migrants in China, particularly in relation to mental health and well-being indicators, including depressive symptoms [6, 11, 31] and subjective well-being [32]. However, there has been limited research delving into the psychological pathway that mediate the link between IEC and the incidence of depression among older internal migrants in the Chinese context. Based on this, we proposed the hypothesis that.

#### H1

IEC is significantly correlated with depression among older internal migrants.

### Potential mediating effect of loneliness

Loneliness is a distressing emotion, typically arising when one’s social needs are inadequately met in terms of both quantity and quality of relationships, particularly in terms quality of interpersonal relationships [33]. It stands as a risk factor for mental health, escalating vulnerability to mental disorders, such as depression [34]. Massive evidence indicates that loneliness serves as a precursor to depressive symptoms among older adults [35–37]. Current research indicates that older internal migrants face an increased risk of loneliness due to changes in their living environment, disruptions to previous social networks, and challenges in social integration [38–40]. Intergenerational relationships have proven to be highly effective in substantially alleviating loneliness among older internal migrants in China [41]. Empirical studies found that intergenerational relationships play a role in diminishing loneliness and subsequent psychological problems among Chinese older parents [19]. Furthermore, the substantial mediating role of loneliness between IEC and depression in older adults has been confirmed in samples settings in China [19, 29, 42]. Consequently, we hypothesize that.

#### H2

Loneliness mediates the relationship between IEC and depression among older internal migrants.

### Potential mediating effect of perceived stress

Perceived stress is an individual’s psychological response to life unpredictability and uncontrollability [43]. One immediate negative consequence of stress is the reduction of mental well-being [44], and it is considered to play a crucial role in the etiology of depression [45]. Research has documented that older adults with a high level of perceived stress are at an increased risk of experiencing depression [46, 47]. With the combination of extrafamilial stressors (i.e., inequal household registration policy, inaccessible basic public health services) [48] and intrafamilial stressors (i.e., acculturation, intergenerational conflicts, role strain from grandparenting) [49, 50], Chinese older internal migrants are facing a high level of stress [51]. Intergenerational relationships and support, being a crucial source of social relations and support, have been recognized as a core factor influencing perceived stress among older adults [52–54]. Moreover, limited studies has substantiated the mediating role of stress between intergenerational relationships and depression [50, 55]. According to the Social Convoy Model, social relations influence the degree of stress an individual experiences, assisting them in coping with stress and enhancing their mental well-being [15, 16]. Thus, we hypothesize that.

#### H3

Perceived stress mediates the relationship between IEC and depression among older internal migrants.

### Potential serial mediating role of loneliness and perceived stress

We are intrigued to investigate the potential relationship between loneliness and perceived stress when both factors are considered as mediators. Loneliness was regarded as a stressor due to its linked perception of social rejection and exclusion [56]. Researchers have validated a positive correlation between loneliness and perceived stress [57]. The connection between loneliness and poor mental health outcomes may be mediated by heightened stress levels reported by lonely individuals. This is because they are less likely to employ adaptive coping mechanisms when confronted with stress compared to non-lonely individuals [58]. Moreover, empirical investigations on in samples settings in China have illustrated that perceived stress serves as a mediator in the association between loneliness and depression among older adults [46, 59]. Integrating Berkman’s theoretical framework, which suggests that social relations influence mental health through psychosocial mechanisms [60], and the Social Convoy Model [15], we developed a hypothesis that.

#### H4

Loneliness and perceived stress play a serial mediating role in the relationship between IEC and depression among older internal migrants.

### The present study

**Fig. 1:**
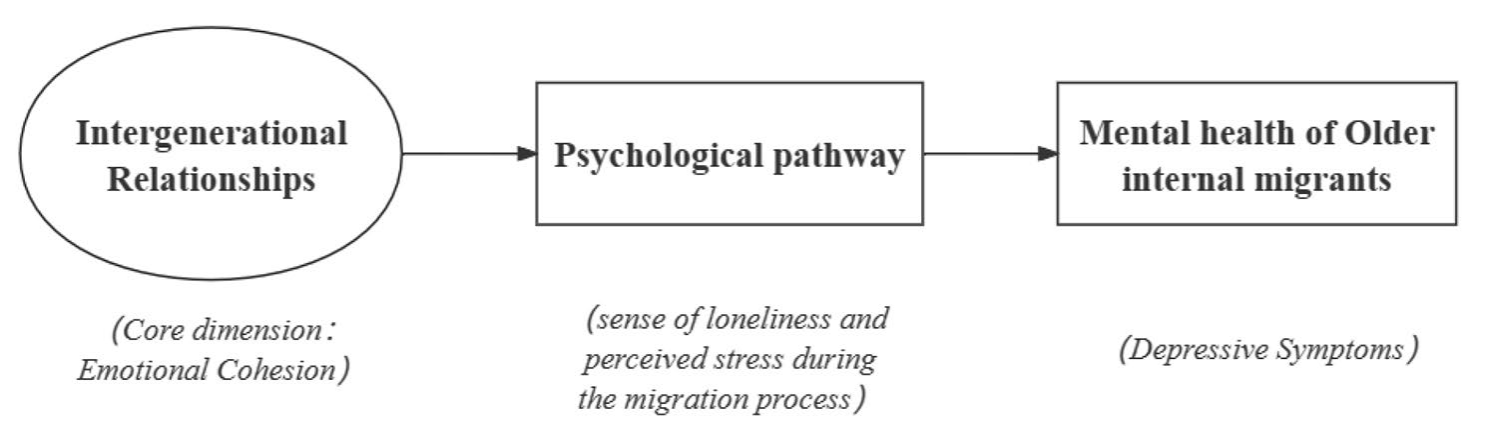
Conceptual framework of psychological pathway linking Intergenerational emotional cohesion to mental health of older internal migrants

**Fig. 2:**
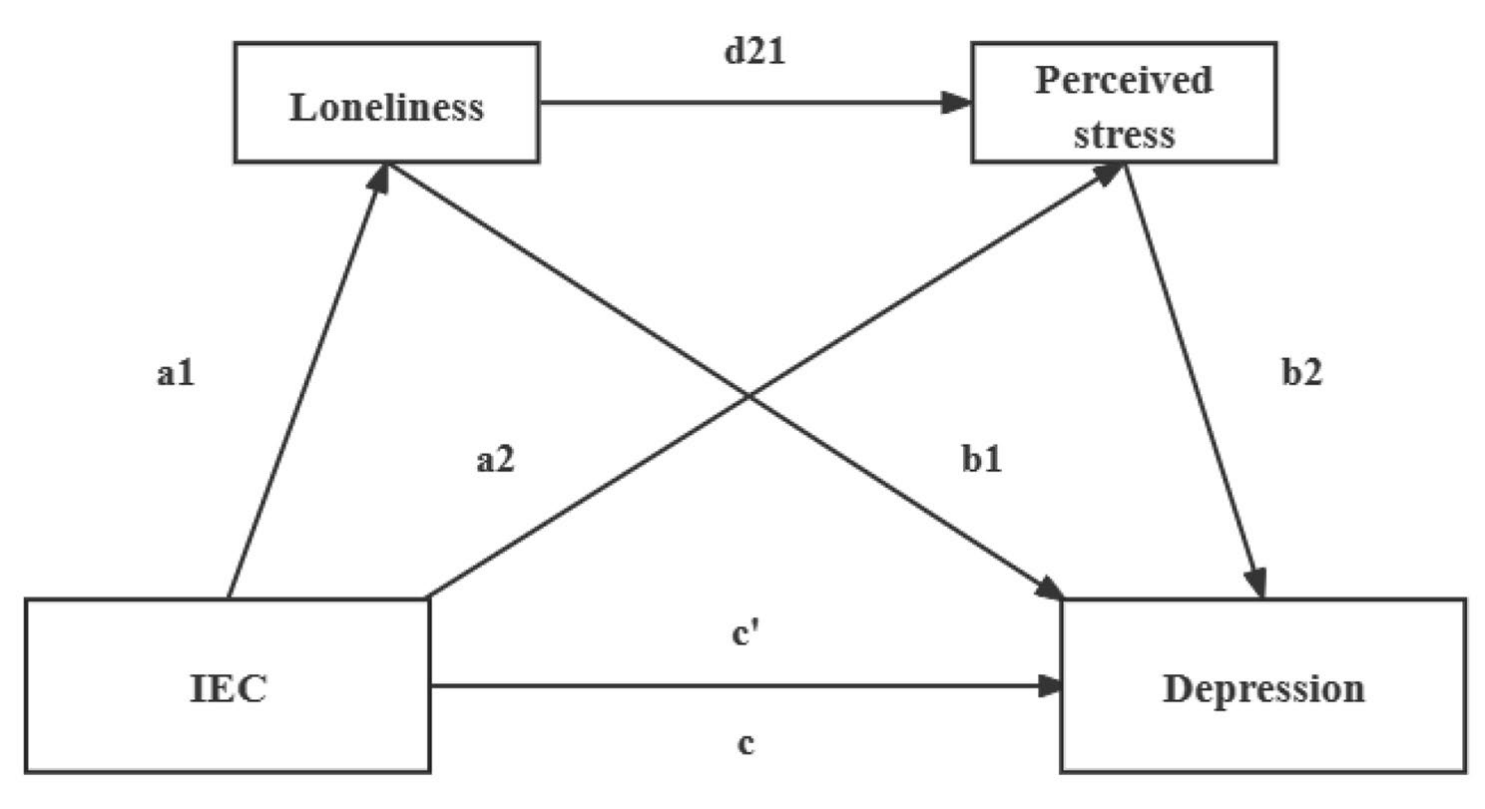
A serial mediation model of the association between IEC and depression through loneliness and perceived stress

This study scrutinizes the pivotal psychological mechanism through which IEC correlates with depression in a sample of older internal migrants within the Chinese context. Drawing on Berkman’s theoretical framework [60] and Social Convoy Model [15], we posit that IEC can impact the depression of older internal migrants by mitigating negative psychological emotions during the migration process. We formulated mediation models with depression as the dependent variable, IEC as the independent variable, and loneliness and perceived stress as mediating factors. The serial mediation mechanism of loneliness and perceived stress in the relationship between IEC and depression was explored. The detailed research framework is illustrated in Fig. 1 and Fig. 2.

## Methods

### Participants

Data for this study were derived from the research project titled ‘A Study on the Mechanism of Intergenerational Relationships on the Mental Health of Older Internal Migrants.’ This cross-sectional investigation transpired in Nanjing, China, spanning from September 2020 to December 2020. Nanjing, as one of China’s supercities experiencing swift economic advancement, exerts significant appeal and absorptive capacity for the migrating population. By 2021, the internal migrant cohort in Nanjing had surged to 2.65 million, denoting an increase of 38.65% since 2010. Consequently, delving into a research inquiry with Nanjing as a focal point provides a high level of representativeness.

The participants were first randomly selected from three communities in the seven districts of Nanjing (Qinhuai, Qixia, Gulou, Xuanwu, Jianye, Yuhuatai, and Jiangning District). Eligibility criteria for inclusion were as follows: (a) age of ≥ 60 years; (b) residency in Nanjing for ≤ 10 years (to capture late-life migration); (c) retention of Hukou in their former residences; and (d) having at least one adult child. Finally, a total of 654 participants were included. All participants engaged in face-to-face interviews employing a structured questionnaire. Each participant was informed of the study details and showed consent to participate in the study. All interviewers, comprised of postgraduate students, possessed prior experience in medical research and underwent standardized training before embarking on the project.

### Measures

#### Intergenerational emotional cohesion

IEC was measured according to the dimensions in the intergenerational solidarity measurement model [27]. Specific measurements were captured by the intergenerational solidarity inventory [61], using the following three questions: (a) ‘Do you think you are emotionally close to your children?’ (no=1, close=2, and very close=3), (b) ‘Do you think you get along well with your children?’ (no=1, well=2, and very well=3), and (c) ‘When talking about your thoughts or difficulties, do you think your children will be willing to listen?’ (no=1, sometimes=2, usually=3). Scores ranges from 3 to 9, with a higher score indicating a better emotional cohesion. Cronbach’s alpha for the present sample was 0.73.

### Loneliness

The 3-item R-UCLA Loneliness Scale [62] was used to measure the loneliness of older internal migrants. It consists of 3 items rated on a 3-point scale (1=‘hardly ever or never’ to 3=‘almost always’), the total score ranges from 3 to 9. A higher score indicates a stronger loneliness. The Chinese version [63] of the scale has proven highly reliable among older adults. Cronbach’s alpha for the present sample was 0.87.

### Perceived stress

Perceived Stress Scale (PSS) [64] was used to measure the perceived stress of older internal migrants over the month earlier. PSS consists of 14 items and two subscales: the sense of uncontrollable and nervous. Each item is scored from 0=‘never’ to 4=‘always’ on the scale, and the total score ranges from 0 to 56. The Chinese version [65] of the scale has shown a high reliability. A higher score indicates a stronger perceived stress. Cronbach’s alpha for the present sample was 0.81.

### Depression

The 9-item Patient Health Questionnaire (PHQ-9) [66] was used to measure the depressive symptoms of older internal migrants over the two weeks before. Nine items were rated on a four-point scale (0=‘never’ to 3=‘almost every day’). The total score ranges from 0 to 27, with a higher value indicating a more severe depression. The Chinese version [67] has been widely used to measure the depressive symptoms among the older adults, with a high reliability. Cronbach’s alpha for the present sample was 0.81. In addition to examining PHQ-9 total scores, we formulated a binary variable with scores ≥ 10 indicating clinically significant depressive symptoms, aligning with published cut-off scores for the PHQ-9 [68].

### Sociodemographic variables

Sociodemographic variables included age, gender (1=‘male’, 2=‘female’), education level (1=‘primary school or lower’, 2=‘junior or senior high school’, 3=‘college or higher’), Hukou (1=‘agricultural’, 2=‘non-agricultural’), marital status (1=‘currently married’, 2=‘widowed’, 3=‘divorced’), living with marital partners (1=‘living with marital partners’, 2=‘living separately from marital partners’), annual income (1= 0-¥5000, 2=¥5001-¥10,000, 3=¥10,001-¥40,000, 4>¥40,000). Subjective health was assessed with the widely used question: ‘How is your present health?’. The answer was given a score ranging from 1=‘very bad’ to 5=‘very good’. Living arrangement (1=‘living with adult children’, 2=‘living separately from adult children’), cause of migration (1=‘grandparenting’, 2=‘enjoying life’, 3=‘working’, 4=‘other’), and duration of migration (years).

### Data analysis

Firstly, we conducted a descriptive analysis to describe the demographic characteristics of the participants. The Chi-square test was employed to explore the relationship between depression and categorical variables, such as gender, education level, Hukou, and others. The independent two-sample t-test was used to scrutinize differences in age, subjective health, and the duration of migration between the depressed and non-depressed groups. Subsequently, intercorrelations among the four key variables (IEC, loneliness, perceived stress, and depression) were examined. Finally, the serial mediation model was performed by using Hayes’ PROCESS macro 3.4 [69]. Paths between mediators were examined by the serial multiple mediator model (PROCESS Model 6). The score of each key variable was standardized (z scores) before analysis. This methodology utilized an ordinary least-square regression model and the bootstrap method to estimate the indirect effect and its 95% confidence intervals (CIs) through random resampling techniques. This approach provides better control over type I errors. The Bootstrap method was employed to assess the significance of these mediation effects using 5000 bootstrap samples [70]. The mediating effect was significant if the bootstrap 95% CIs did not include zero [71]. Furthermore, age, gender, education level, hukou, marital status, living with marital partners, annual income, subjective health, living arrangement, cause of migration, as well as duration of migration were introduced into the model as covariates. The statistical analysis was conducted using the IBM SPSS 25.0 software, and the significance level was set at 0.05 (two-tailed).

## Results

### Sample profile and correlation analysis

Table 1 presents the demographic characteristics of all 654 participants. In this study, the mean age of older internal migrants was 66.05 years (SD=4.67, range=60-86). The mean score for subjective health was 3.07 (SD=0.94, range=1-5). The average duration of migration in Nanjing was 3.96 years (SD=1.46, range=0.10-5.50). Over two-thirds of the participants were female (67.00%). More than half of them had an education level of primary school or lower (54.13%), and 69.27% had an agricultural Hukou. Four-fifths of the participants were married (84.56%), and half of them lived with their marital partners (51.22%). Over half of the participants reported an annual income lower than ¥10,000 (57.34%), and the majority were living with their adult children (83.94%). Additionally, 77.37% migrated to take care of their grandchildren.

**Table 1:** Demographic characteristics and measurements of participants (*n* = 654)

Variables	Category	Mean (SD) /N (%)
Age		66.05 (4.67)
Gender	Male	216 (33.03%)
	Female	438 (66.97%)
Education level	Primary school or lower	354 (54.13%)
	Junior or senior high school	262 (40.06%)
	College or higher	38 (5.81%)
Hukou	Agricultural	453 (69.27%)
	Non-agricultural	201 (30.73%)
Marital status	Currently married	553 (84.56%)
	Widowed	90 (13.76%)
	Divorced	11 (1.68%)
Living with marital partners	Living with marital partners	335 (51.22%)
	Living separately from marital partners	319 (48.78%)
Annual income	0-¥5000	249 (38.07%)
	¥5001-¥10,000	126 (19.27%)
	¥10,001-¥40,000	199 (30.43%)
	>¥40,000	80 (12.23%)
Subjective health		3.07 (0.94)
Living arrangement	Living with adult children	549 (83.94%)
	Living separately from adult children	105 (16.06%)
Cause of migration	Grandparenting	506 (77.37%)
	Enjoying life	86 (13.15%)
	Working	19 (2.91%)
	Other	43 (6.57%)
Duration of migration		3.96 (1.46)

Table 2 illustrates the association of depression with participants’ demographic characteristics. The majority of participants had a PHQ-9 score less than 10 (88.23%). When categorical groups were compared within the PHQ-9 depression group (< 10 versus ≥ 10), there was a statistically significant difference between gender (p-value = 0.097), Hukou (*p*-value=0.080), marital status (*p*-value=0.033), and cause of migration (*p*-value=0.020) concerning the presence of depressive symptoms. Additionally, a statistically significant difference in age distribution across the depression groups was observed (*p*-value=0.060).

**Table 2:** Association of depression with participants demographic characteristics (*N* = 654)

Variables	Category	PHQ-9 (< 10)	PHQ-9 (≥ 10)	*p*-value
Age		66.15 (4.59)	65.35 (5.21)	0.066^†^
Gender	Male	197 (91.20%)	19 (8.80%)	0.097^†^
	Female	380 (86.76%)	58 (13.24%)	
Education level	Primary school or lower	308 (87.01%)	46 (12.99%)	0.508
	Junior or senior high school	234 (89.31%)	28 (10.69%)	
	College or higher	35 (92.11%)	3 (7.89%)	
Hukou	Agricultural	393 (86.75%)	60 (13.25%)	0.080^†^
	Non-agricultural	184 (91.54%)	17 (8.46%)	
Marital status	Currently married	495 (89.51%)	58 (10.49%)	0.033*
	Widowed	72 (80.00%)	18 (20.00%)	
	Divorced	10 (90.91%)	1 (9.09%)	
Living with marital partners	Living with marital partners	300 (89.55%)	35 (10.45%)	0.281
	Living separately from marital partners	277 (86.83%)	42 (13.17%)	
Annual income	0-¥5000	219 (87.95%)	30 (12.05%)	0.415
	¥5001-¥10,000	107 (84.92%)	19 (15.08%)	
	¥10,001-¥40,000	177 (88.94%)	22 (11.06%)	
	>¥40,000	74 (92.50%)	6 (7.50%)	
Subjective health		3.05 (0.94)	3.18 (0.91)	0.630
Living arrangement	Living with adult children	482 (87.80%)	67 (12.20%)	0.435
	Living separately from adult children	95 (90.48%)	10 (9.52%)	
Cause of migration	Grandparenting	457 (90.32%)	49 (9.68%)	0.020*
	Enjoying life	71 (82.56%)	15 (17.44%)	
	Working	15 (78.95%)	4 (21.05%)	
	Other	34 (79.07%)	9 (20.93%)	
Duration of migration		3.99 (1.45)	3.70 (1.54)	0.111

Note: Values are given as ‘Mean (SD)’ or ‘N (%)’, ^†^*p* < 0.10, *p < 0.05

Table 3 shows the Spearman correlation coefficients for all variables. IEC was IEC exhibited a significant negative correlation with loneliness (*r*=-0.26, *p* < 0.01), perceived stress (*r*=-0.30, *p* < 0.01), and depression (*r*=-0.36, *p* < 0.01). Loneliness exhibited a positive correlation with perceived stress (*r*=0.40, *p* < 0.01) and depression (*r*=0.45, *p* < 0.01). Furthermore, perceived stress showed positive correlation with depression (*r*=0.54, *p* < 0.01).

**Table 3:** Spearman correlation analysis among the key variables (*N* = 654)

Variables	Mean	SD	Range	1	2	3	4
1 IEC	7.75	1.34	3–9	1			
2 Loneliness	4.02	1.43	3–9	− 0.26**	1		
3 Perceived Stress	20.87	7.66	15–70	− 0.30**	0.40**	1	
4 Depression	5.01	3.99	0–27	− 0.36**	0.45**	0.54**	1

Note: IEC: intergenerational emotional cohesion. *p < 0.05, **p < 0.01(two-tailed)

### Serial mediation model results

**Fig. 3:**
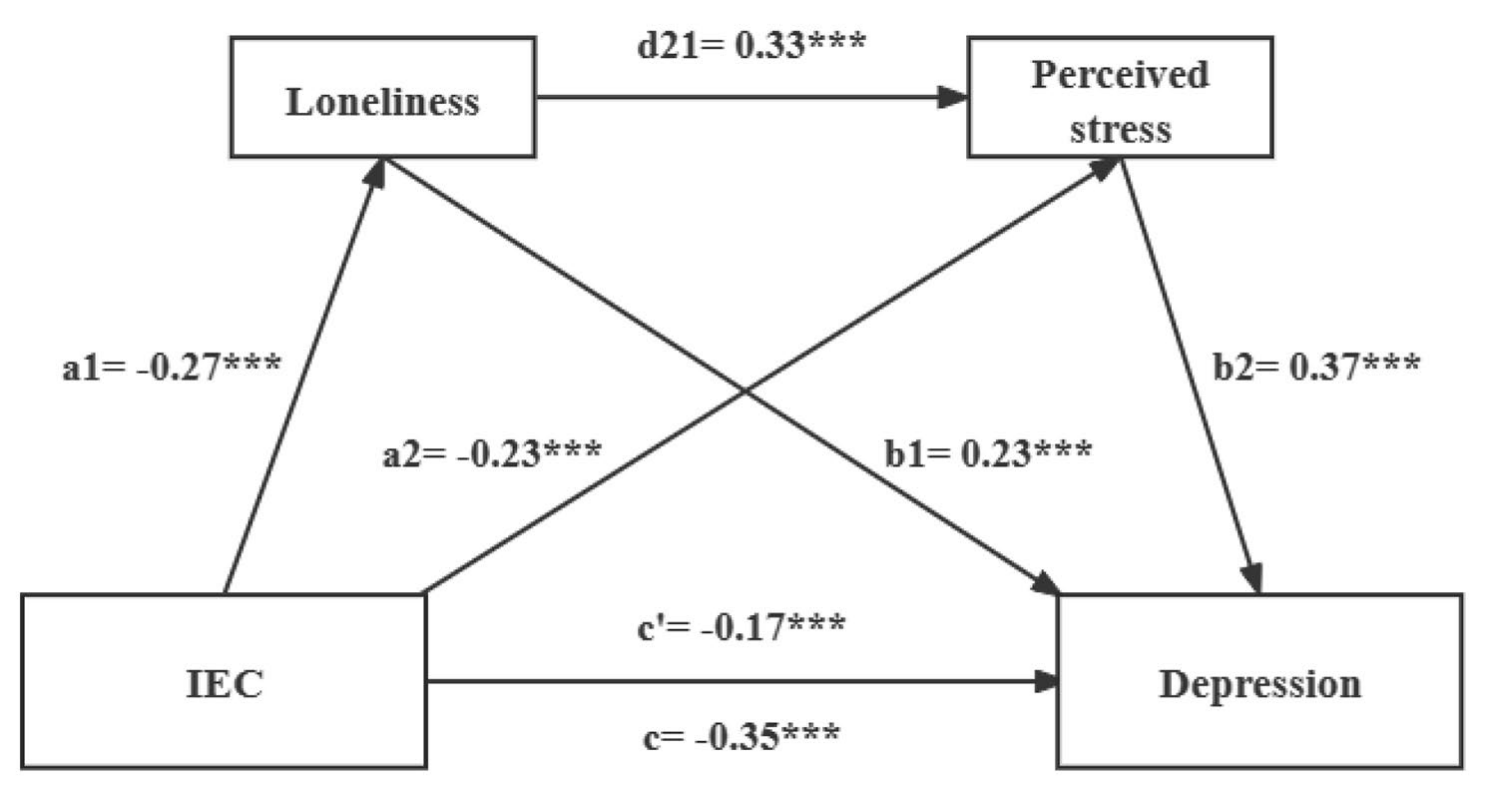
A serial mediation model of the association between IEC and depression through loneliness and perceived stress. Standardized path coefficients are shown. Note: IEC: intergenerational emotional cohesion, *p < 0.05, **p < 0.01, ***p < 0.001

In the serial mediation analysis, IEC was entered as an independent variable and depression as a dependent variable. Loneliness and perceived stress were proposed as mediators. As shown in Table 4; Fig. 3, IEC significantly and negatively predicted depression (c’=-0.17, *p* < 0.001), thus confirming **H1**. As the regression equation included loneliness and perceived stress, IEC exhibited a significant negative effect on loneliness (a1=-0.27, *p* < 0.001) and perceived stress (a2=-0.23, *p* < 0.001). Loneliness had a significant positive effect on perceived stress (d21=0.33, *p* < 0.001) and depression (b1=0.23, *p* < 0.001). Moreover, perceived stress was a significant positive predictor of depression (b2=0.37, *p* < 0.001). These results indicated that the serial mediating effect of ‘loneliness → perceived stress’ was significant among the influences of IEC on depression, thus confirming **H2**, **H3** and **H4**.

**Table 4:** Serial mediation analysis among the key variables (*N* = 654)

Result variable	Predictor variable	R	R^2^	F	B	t	95%CI
Loneliness	IEC	0.30	0.09	5.75	-0.27***	-7.20	(-0.35, -0.20)
perceived stress	IEC	0.49	0.24	17.11	-0.23***	-6.48	(-0.30, -0.16)
	Loneliness	-	-	-	0.33***	9.09	(0.26, 0.40)
Depression	IEC	0.60	0.36	27.81	-0.17***	-4.84	(-0.23, -0.10)
	Loneliness	-	-	-	0.23***	6.57	(0.16, 0.30)
	Perceived stress	-	-	-	0.37***	10.04	(0.29, 0.44)

Note: IEC: intergenerational emotional cohesion, *p < 0.05, **p < 0.01, ***p < 0.001; Age, gender, Hukou, marital status, living arrangement, education level, and annual income were analyzed as control variables

### Bootstrap of mediators

The mediation effects of loneliness and perceived stress are presented in Table 5. Loneliness and perceived stress mediated the relationship between IEC and depression, with a mediating effect value of -0.18 that accounted for 51.43% of the total effect. Specifically, the indirect effect contained three mediation pathways: (1) path 1: IEC →loneliness → depression (β=-0.06, 95% CI=-0.10, -0.04), (2) path 2: IEC → perceived stress → depression (β=-0.09, 95% CI=-0.12, -0.05), (3) path 3: IEC → loneliness → perceived stress → depression (β=-0.03, 95% CI=-0.05, -0.02). The three indirect effects accounted for 17.14%, 25.71%, and 8.57% of the total effect, respectively. Each of them reached a significant level, because the 95% CI of the above indirect effects did not contain zero. These results further supported** H2**, **H3** and **H4**.

**Table 5:** The indirect effects of IEC on depression with loneliness and perceived stress as mediators (*N* = 654)

Model pathways	B	Boots SE	Mediating effect	95%CI	
				Lower	Upper
Total effects	-0.35^a^	0.04	100%	-0.42	-0.27
Direct effects	-0.17^a^	0.03	48.57%	-0.23	-0.10
Indirect effects	-0.18^a^	0.03	51.43%	-0.23	-0.13
path1: IEC → Loneliness → Depression	-0.06^a^	0.02	17.14%	-0.10	-0.04
path2: IEC → Perceived stress → Depression	-0.09^a^	0.02	25.71%	-0.12	-0.05
path3: IEC → Loneliness → Perceived stress → Depression	-0.03^a^	0.01	8.57%	-0.05	-0.02

Note: IEC: intergenerational emotional cohesion. ^a^ The bootstrap 95% CIs not contain zero

## Discussion

This study explores the substantial impact of IEC on depression among older internal migrants in mainland China and examines the potential mediating effects of loneliness and perceived stress. As anticipated, the results revealed that IEC played a role in decreasing depression both directly and indirectly through the serial mediating effects of loneliness and perceived stress. The key findings of this study are detailed below.

First, correlational and regression analyses conducted in the study revealed that IEC significantly and negatively predicted depression. This implies that IEC is associated with a reduced likelihood of depression among Chinese older internal migrants, supporting hypothesis H1. This finding aligns with previous empirical studies suggesting that emotional cohesion can mitigate depressive symptoms and enhance mental well-being among older migrants [72, 73].This phenomenon can be explained by the Social Convoy Model [15] and the socioemotional selectivity theory [74]. Older internal migrants, facing the challenges of aging and social integration, tend to seek affective closeness from their closest family members, enhancing positive emotional experiences and minimizing mental health risks [17]. Therefore, IEC emerges as a crucial factor in preventing and alleviating depression among older internal migrants.

Second, the investigation unveiled that loneliness mediates the association between IEC and depression, with an impact of 18.44%, substantiating hypothesis H2. IEC can predispose older parents to experience less depression by alleviating feelings of loneliness during the migration process. This finding aligns with antecedent research, signifying that familial intimacy functions as a safeguard against depression by mitigating feelings of loneliness [19]. Consistent with attachment theory [75], the reliance of older internal migrants on their adult children assumes a pivotal role. On one hand, the provision of emotional support by adult children effectively alleviates loneliness [76, 77]. On the other hand, social integration is bolstered through the social support extended by adult children, fostering an enhanced sense of belonging and diminished loneliness among older internal migrants [39]. Consequently, subsequent mental health challenges such as psychological stress and depression are more aptly alleviated [78].

Third, we observed that perceived stress was a robust mediator between IEC and depression, with an effect of 24.58%. Therefore, H3 was supported. Although perceived stress has been linked to intergenerational relationships and depression [34], few studies have explored its mediating role. In this study, our data shed light into the mediating role of perceived stress in IEC-depression association among immigrant parents. For Chinese olde adults, migration is usually accompanied by a range of intrafamilial stressors, such as acculturation, family conflicts, role strain from grandparenting [49, 50], each of which may cause psychological distress. Aligned with the Social Convoy Model [15, 16], IEC, serving as the core social relations among older internal migrants, proves advantageous in mitigating various stressors and enhancing their capacity to cope with stressful events, thereby promoting their mental well-being. This could elucidate the comparatively higher mediating effect of perceived stress.

Additionally, in the current study, loneliness and perceived stress played a serial mediating role in the association between IEC and depression, with an effect of 9.47%. Therefore, hypothesis H4 found support. IEC can influence the depression of older internal migrants by alleviating loneliness and perceived stress during the migration process. According to the added-stress hypothesis [79], loneliness, as a stressor associated with perceptions of social rejection and exclusion, heightens individuals’ vulnerability to life stress events and weakens their resilience against them. Additionally, perceived stress and loneliness have been identified as contributory factors to depression among older adults [80]. Consistent with Berkman’s theoretical framework [60] and the Social Convoy Model [15], the pathway through which intergenerational relationships influence mental health via psychological mechanisms can be deduced as ‘IEC → loneliness → perceived stress → depression.’ This finding contributes novel insights to the literature on mental health among older internal migrants.

Several limitations of this study need to be acknowledged. Firstly, it relies on cross-sectional data, limiting the ability to establish causality between the core variables. Besides, reverse causality might impact the study’s robustness. Future research with a longitudinal design is recommended to explore causal relationships. Secondly, due to limitations in the survey design, we lacked an objective assessment of the physical health status of older internal migrants, such as the prevalence of chronic diseases and the activity of daily living (ADL). Future research should consider including these variables to control for the effects of physical health status on depression in older adults. Lastly, intergenerational relationships among older internal migrants may differ from those of left-behind older parents and non-migrant individuals. A comparative analysis of these three groups could provide deeper insights into intergenerational relationships and their effects on the mental health of older internal migrants.

## Conclusions

Despite its limitations, this study delves into the impact of intergenerational relationships on depression among older internal migrants, exploring the psychological mechanisms within familistic cultures and migration contexts. Based on the Berkman’s theoretical framework and the Social Convoy Model, the findings suggest that IEC is linked to depression, both directly and indirectly. More precisely, it can influence depression by alleviating loneliness and perceived stress among older internal migrants. In summary, these results contribute to existing studies on intergenerational relationships and depression among older internal migrants, offering theoretical guidance for preventing mental health problems.

## Data Availability

The datasets used and/or analysed during the current study are available from the corresponding author on reasonable request.
